# Supplementary material for: IL-20 Activates ERK1/2 and Suppresses Splicing of X-Box Protein-1 in Intestinal Epithelial Cells but Does Not Improve Pathology in Acute or Chronic Models of Colitis
Source: Int J Mol Sci. 2022 Dec 22;24(1):174. doi: 10.3390/ijms24010174 (PMC9820550; doi:10.3390/ijms24010174)
Supplement: Supplementary file 1 [file ijms-24-00174-s001.zip › ijms-2057047-supplementary.pdf]

Supplementary Figure S1

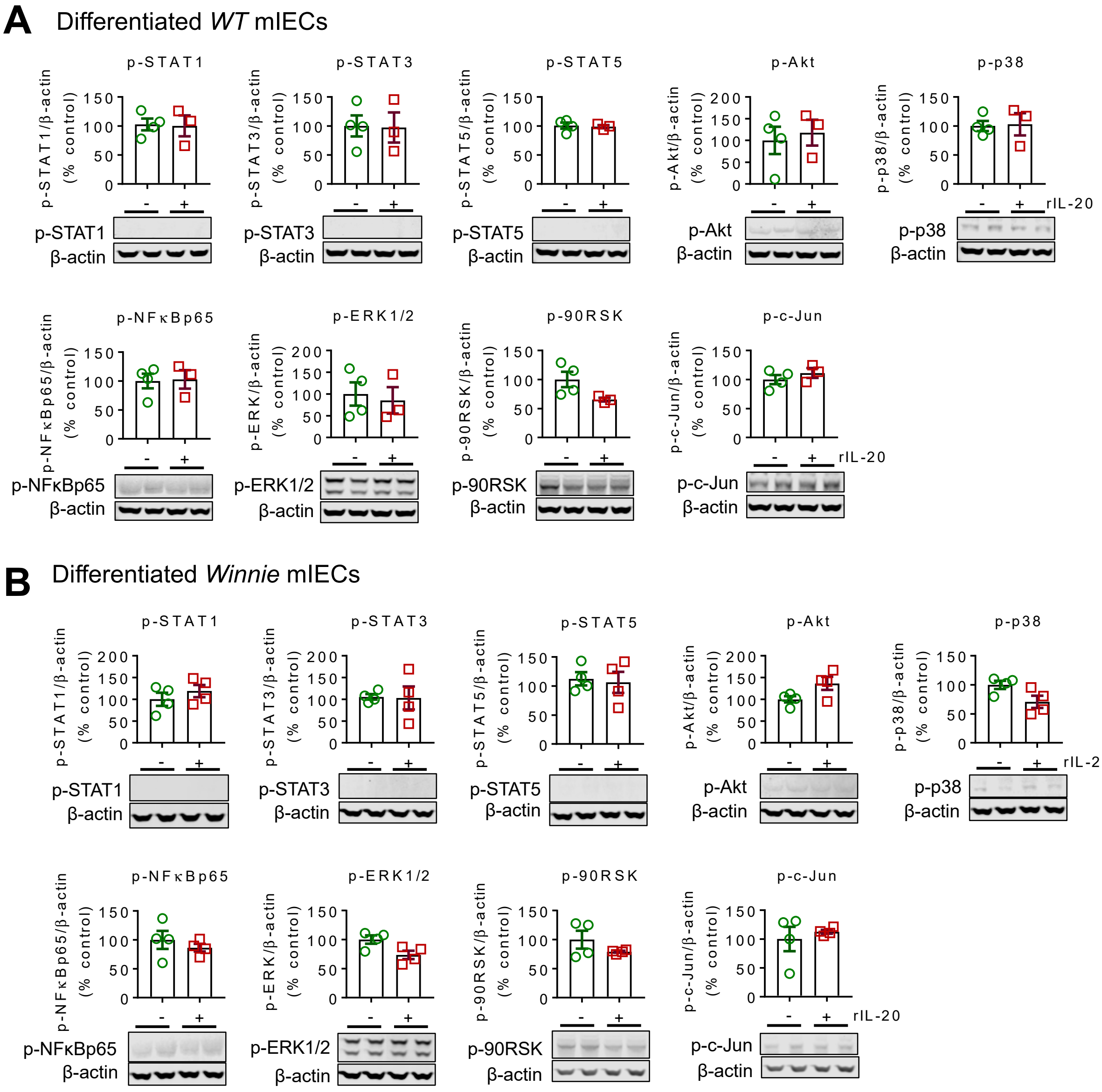

Figure S1: rIL-20 specific modulation of signalling pathways in differentiated primary intestinal epithelial cells. DAPT treated primary colonic organoids from C57BL/6 (A) and Winnie (B) were analysed for phosphorylated STAT1, STAT3, STAT5, Akt, p38, NF-kβ p65, ERK1/2, 90-RSK, and p-c-Jun by Western blot assay after treatment with rIL-20 (100 ng/mL) for 1 h. Data are presented as mean ± SEM with individual cultures from 3-4 independent experiments (non-parametric Man-Whitney t-test).

Supplementary Figure S2

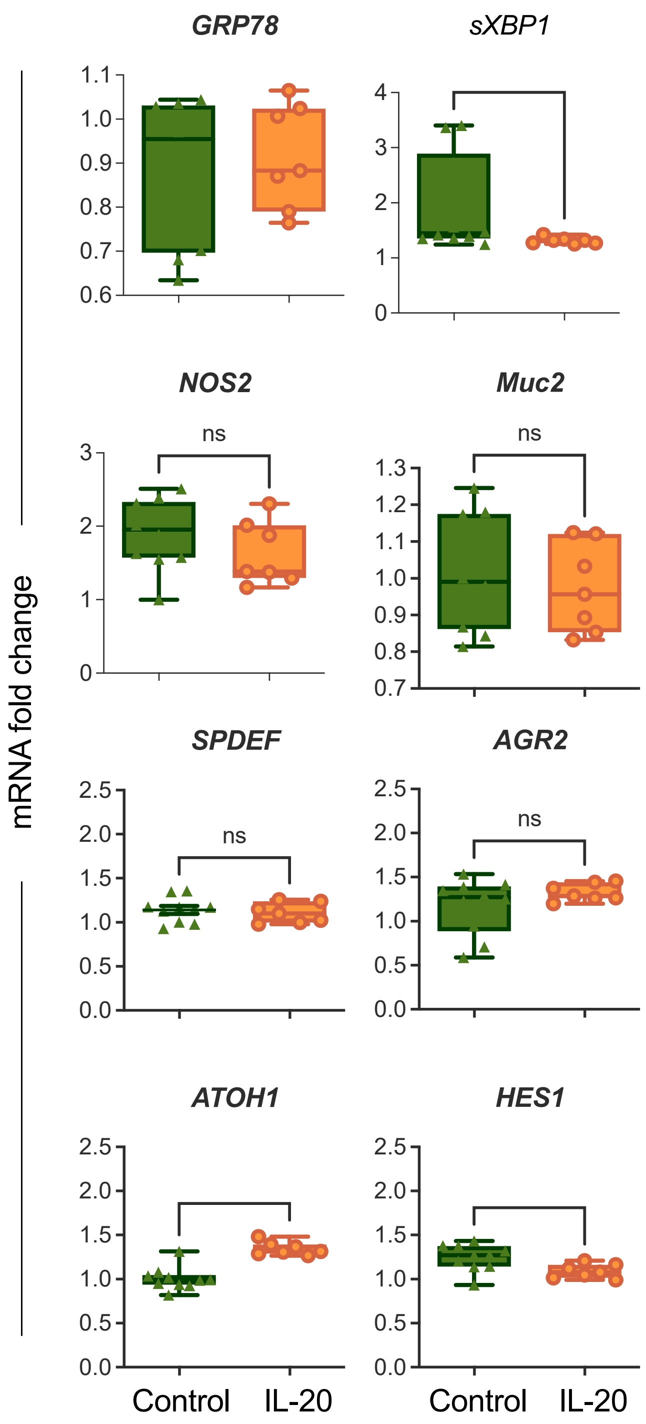

Figure S2: Intestinal cellular stress and differentiation markers in the rIL-20 treated LS174T cells. Relative mRNA expression levels of ER stress, goblet cell and intestinal stem cell inducing transcriptional factors measured by qRT-PCR in the LS174T cells treated with vehicle (PBS) or IL-20 (100 ng/mL) for 24 h. Data are presented as mean ± SEM (n=7-9), compared with PBS controls, student's t-test.

Supplementary Figure S3

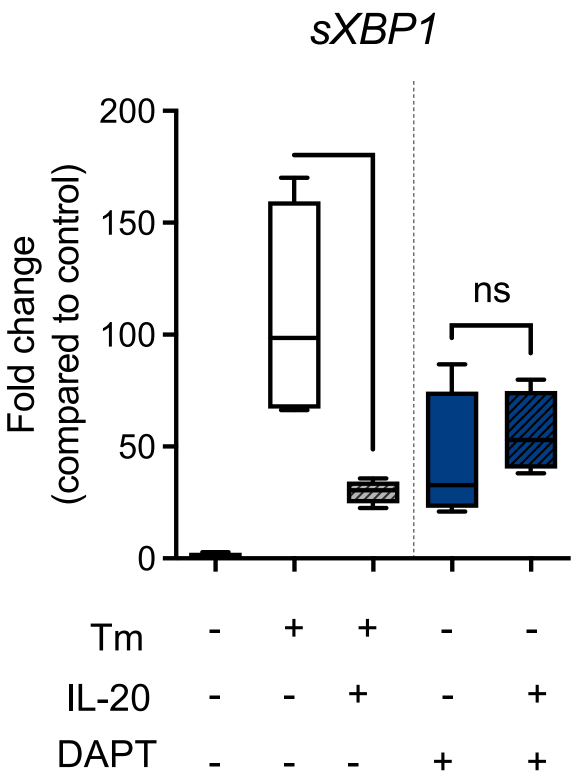

Figure S3: rIL-20 modulates cellular ER stress in LS174T cell lines. Undifferentiated and differentiated LS174T cell lines were co-treated with 10 µM tunicamycin (6 h) and rIL-20 (100 ng/mL) for 24 h and analysed for cellular stress marker, *sXBP1* by qRT-PCR. Data are presented as mean ± SEM (n=12), *P<0.01, one-way ANOVA followed by Dunnett's post hoc test.

Supplementary Figure S4

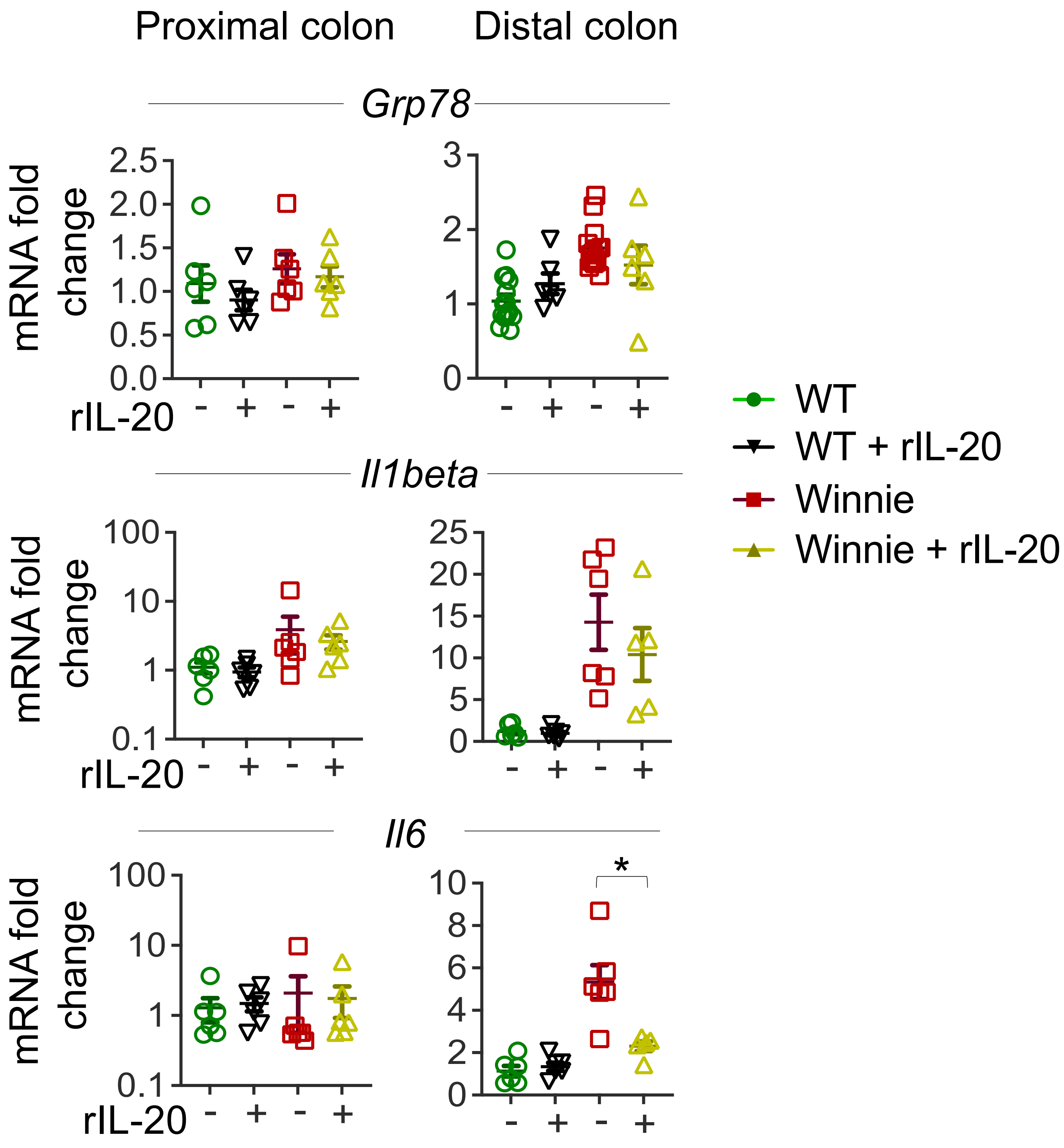

Figure S4: ER stress and pro-inflammatory markers in rIL-20 treated Winnie mice. Relative mRNA levels of *GRP78*, *Il1β* and *Il6* measured by qRT-PCR in the proximal and distal colon tissues harvested from naïve and rIL-20 treated C57BL/6 and Winnie mice (as described in Figure 3). Data are presented as mean ± SEM (n=6-8). * P<0.05, one-way ANOVA followed by Dunnett's post hoc test.

Table S1: Primer Sequences

Primers	Sequence
mTata box	Forward: CTCAGTTACAGGTGGCAGCA
	Reverse: ACCAACAATCACCAACAGCA
mGrp78	Forward: TGCTGCTAGGCCTGCTCCGA
	Reverse: CGACCACCGTGCCCACATCC
m/hXbp1	Forward: GAGTCCGCAGCAGGTGC
	Reverse: CAAAAGGATATCAGACTCAGAATCTGAA
mNos2	Forward: CAGCTGGGCTGTACAAACCTT
	Reverse: CATTGGAAGTGAAGCGTTTCG
mMuc2	Forward: CCATTGAGTTTGGGAACATGC
	Reverse: TTCGGCTCGGTGTTCAGAG
mAgr2	Forward: CGGTGAGGGCAGACATCACTGGA
	Reverse: CCGGTGCGCAGTTGGCTCTA
mHes1	Forward: GGTCCTAACGCAGTGTCACC
	Reverse: GAGAGGTGGGCTAGGGACTT
mSpdef	Forward: GGTGCCTGCTACTGTTCCCAGATG
	Reverse: AAAGCCACTTCTGCACGTTACCAG
mAtoh-1	Forward: GCTTCCTCTGGGGGTTACTC
	Reverse: GAAGGCGACAGGTCCTTCTG
mIL-1β	Forward: CAACCAACAAGTGATATTCTCCATG
	Reverse: GATCCACACTCTCCAGCTGCA
mTnf-α	Forward: CATCTTCTCAAAATTCGAGTGACAA
	Reverse: TGGGAGTAGACAAGGTACAACCC
mIL-6	Forward: TCAGGAAATTTGCCTATTGAAA
	Reverse: GGAAATTGGGGTAGGAAGGA
mMip2α	Forward: ACGTGTTCCAGGACACAACA
	Reverse: ACAAACCCTCCCCACCTAAC
mIL-10	Forward: AGCTCCAAGACCAAGGTGTC
	Reverse: TCCAAGGAGTTGTTTCCGTTA
mIL-17a	Forward: CTCCAGAAGGCCCTCAGACTAC
	Reverse: AGCTTTCCCTCCGCATTGACACAG
mIL-23p19	Forward: AGCGGGACATATGAATCTACTAAGAGA
	Reverse: GTCCTAGTAGGGAGGTGTGAAGTTG
Reg3β	Forward: ACTCCCTGAAGAATATACCCTCC
	Reverse: CGCTATTGAGCACAGATACGAG
Reg3γ	Forward: GGCCATATCTGCATCATACCAG
	Reverse: ATGCTTCCCCGTATAACCATCA
mLrg1	Forward: TTGGCAGCATCAAGGAAGC
	Reverse: CAGATGGACAGTGTCGGCA
mTimp1	Forward: TACACCCCAGTCATGGAAAGC
	Reverse: CGGCCCGTGATGAGAAACT
mTrim15	Forward: TGAGCGAGACCTACTGTGAAG

	Reverse: AACCGACTCCTGAGACGATCC
mOsmr	Forward: TCAAGCCACGAAGGGTCCTAA
	Reverse: GTCTTAAAGTCTCGGGTTTCACA
mLcn2	Forward: TGGCCCTGAGTGTCATGTG
	Reverse: CTCTTGTAGCTCATAGATGGTGC
mIL-18	Forward: ACGTGTTCCAGGACACAACA
	Reverse: ACAAACCCTCCCCACCTAAC
mIL-33	Forward: CGGATCCACTTCACTTTTAACACAGTC
	Reverse: GAGATCTTTAGATTTTCGAGAGCTTA
mIL-13ra1	Forward: AGCGTCTCTGTCGAAAATCTCT
	Reverse: GAGTGCAATTTGGACTGGCTC
mSmad4	Forward: ACACCAACAAGTAACGATGCC
	Reverse: GCAAAGGTTTCACTTTCCCCA
mSocs3	Forward: GGACCAAGAACCTACGCATCCA
	Reverse: CACCAGCTTGAGTACACAGTCG
mClaudin4	Forward: GGCGTCTATGGGACTACAGG
	Reverse: GAGCGCACAACTCAGGATG
mClaudin8	Forward: GTGGATGTGGCCCTAAAGC
	Reverse: CGCTGTGGTCCAGCCTAT
mZo-1	Forward: TTTTTGACAGGGGGAGTGG
	Reverse: TGCTGCAGAGGTCAAAGTTCAAG
